# IgA Anti-β2-Glycoprotein I Autoantibodies Are Associated with an Increased Risk of Thromboembolic Events in Patients with Systemic Lupus Erythematosus

**DOI:** 10.1371/journal.pone.0012280

**Published:** 2010-08-19

**Authors:** Nadera J. Sweiss, Ronghai Bo, Reena Kapadia, Deborah Manst, Farzan Mahmood, Tara Adhikari, Suncica Volkov, Maria Badaracco, Mary Smaron, Anthony Chang, Joseph Baron, Jerrold S. Levine

**Affiliations:** 1 Section of Rheumatology, Department of Medicine, University of Chicago, Chicago, Illinois, United States of America; 2 Section of Rheumatology, Department of Medicine, University of Southern California, Los Angeles, California, United States of America; 3 Loyola University Chicago Stritch School of Medicine, Maywood, Illinois, United States of America; 4 Division of Pulmonary and Critical Care, University of Cincinnati Medical Center, Cincinnati, Ohio, United States of America; 5 Section of Rheumatology, Department of Medicine, University of Illinois at Chicago, Chicago, Illinois, United States of America; 6 Department of Pathology, University of Chicago, Chicago, Illinois, United States of America; 7 Sections of Hematology-Oncology, Department of Medicine, University of Chicago, Chicago, Illinois, United States of America; 8 Section of Nephrology, Department of Medicine, University of Illinois at Chicago, Chicago, Illinois, United States of America; 9 Section of Nephrology, Department of Medicine, Jesse Brown Veterans Affairs Medical Center, Chicago, Illinois, United States of America; New York University, United States of America

## Abstract

**Background:**

The clinical utility of testing for antiphospholipid antibodies (aPL) of IgA isotype remains controversial.

**Methodology/Principal Findings:**

To address this issue, we reasoned that if IgA aPL contribute to the clinical manifestations of the antiphospholipid syndrome, then an association with thromboembolic events should manifest in patients whose only aPL is of IgA isotype. We performed a retrospective chart review of 56 patients (31 with systemic lupus erythematosus [SLE] and 25 without SLE) whose only positive aPL was IgA anti-β2-glycoprotein I (isolated IgA anti-β2GPI) and compared their clinical features with 56 individually matched control patients without any aPL. Patients with isolated IgA anti-β2GPI had a significantly increased number of thromboembolic events, as compared to controls. When patients were stratified into those with and without SLE, the association between isolated IgA anti-β2GPI and thromboembolic events persisted for patients with SLE, but was lost for those without SLE. Titers of IgA anti-β2GPI were significantly higher in SLE patients who suffered a thromboembolic event. Among patients with isolated IgA anti-β2GPI, there was an increased prevalence of diseases or morbidities involving organs of mucosal immunity (i.e., gastrointestinal system, pulmonary system, and skin).

**Conclusions/Significance:**

The presence of isolated IgA anti-β2GPI is associated with an increased risk of thromboembolic events, especially among patients with SLE. IgA anti-β2GPI is associated with an increased prevalence of morbidities involving organs of mucosal immunity.

## Introduction

The clinical utility of testing for antiphospholipid antibodies (aPL) of IgA isotype remains controversial. According to the international consensus statement on antiphospholipid syndrome (APS), aPL of IgA isotype (either anticardiolipin antibodies [aCL] or anti-β2-glycoprotein I antibodies [anti-β2GPI]) do not fulfill laboratory criteria for APS classification [1]. This exclusion was based on the opinion that these autoantibodies lack specificity and do not provide independent clinical information from that given by IgM or IgG aPL. However, in certain cohorts, many individuals test positive for IgA aPL alone or with other aPL [1]–[18]. Patients with IgA aPL only are particularly important, as they cannot be classified as having APS even when manifesting APS symptoms.

To determine whether IgA aPL are associated with clinical manifestations, we used an approach that differs from previous studies. We reasoned that if IgA aPL contribute to APS manifestations, then thromboembolic events should manifest in patients with isolated IgA aPL. These patients do not manifest any other aPL, including non-IgA aPL or lupus anticoagulant (LA) activity, associated with APS. Therefore, we performed a retrospective chart review of patients with isolated IgA anti-β2GPI and compared their clinical features with individually matched controls to determine whether isolated IgA anti-β2GPI is associated with an increased risk for thromboembolic events, especially within a background of systemic lupus erythematosus (SLE).

## Materials and Methods

### Ethics

The medical records of patients and controls were reviewed according to a study protocol approved by the University of Chicago Institutional Review Board. Patient consent was waived, as this was a retrospective chart review and no therapeutic interventions were involved.

### Participants

We performed a retrospective review of the laboratory results of all patients who underwent complete aPL testing at the University of Chicago Hospital between November, 2001, and July, 2007. Complete aPL testing was defined as including all of the following: aCL (IgM, IgG, and IgA); anti-β2GPI (IgM, IgG, and IgA); and LA (activated partial thromboplastin time [aPTT], dilute Russell viper venom time [dRVVT], and tissue thromboplastin inhibition test [TTI]). Of 5602 patients who underwent complete aPL testing, we identified 56 (1%) whose only positive aPL was IgA anti-β2GPI: 31 patients with SLE and 25 patients without SLE. It should be emphasized that these 56 patients with an isolated IgA anti-β2GPI (hereafter called “isolated IgA anti-β2GPI”) were negative for IgA aCL. An additional 21 of the 5602 patients were positive for IgA anti-β2GPI, but had concomitant elevation of one or more other aPL and were therefore not included (5 IgM aCL; 2 IgG aCL; 3 IgA aCL; 2 IgM plus IgA aCL; 2 IgG plus IgA aCL; 1 IgM plus IgG aCL; 2 IgM, IgG, plus IgA aCL; and 4 LA). No patients who underwent complete aPL testing or who had isolated IgA anti-β2GPI were excluded. Controls were individuals who underwent complete aPL testing and were negative for all aPL. They were individually matched to patients based on age, gender, race, and disease status (32 with and 24 without SLE). SLE was defined according to American College of Rheumatology (ACR) criteria [19]. As patients in our study were positive for IgA anti-β2GPI, but negative for all other aPL, SLE status was unchanged by application of the revised ACR criteria, which include aPL positivity [20].

### Clinical data

Clinical data at baseline included demographic parameters (age, gender, and race); general comorbidities (hypertension and diabetes mellitus); SLE status; history of pregnancy morbidity; occurrence of arterial, venous, and/or microvascular thromboembolic events; medication history; and comorbidities of organs or tissues involved in mucosal immunity (gastrointestinal [GI] system, pulmonary system, or skin).

Arterial events included cerebrovascular accident (CVA), transient ischemic attack (TIA), myocardial infarction (MI), angina, or other acute events involving the arterial vasculature. Venous events included deep vein thrombosis (DVT), pulmonary embolism (PE), or other acute events involving the venous vasculature. Microvascular events included biopsy-proven thrombotic microangiopathy (TMA). Criteria for thromboembolic events included confirmatory diagnostic tests and/or documented clinical diagnoses by treating physicians. Patients had no previous medical history of thrombosis, as confirmed by the treating physician and documented in physician notes. Only confirmed events were used in our analyses. Criteria for comorbidities involving the GI system, pulmonary system, or skin included confirmatory diagnostic tests and/or documented clinical diagnoses by treating physicians. Specific etiologies for comorbidities were not required.

### aPL assays

All testing for aPL was performed by the University of Chicago clinical laboratories, according to instructions by the assay's manufacturer. Levels of anti-β2GPI were determined by ELISA using separate QUANTA Lite Test Kits for IgG, IgM, and IgA isotypes (B2-GPI IgG ELISA, B2-GPI IgM ELISA, and B2-GPI IgA ELISA; INOVA Diagnostics, Inc., San Diego, CA). Levels of aCL were determined by ELISA using QUANTA Lite Test Kits for IgG, IgM, and IgA isotypes (ACA IgM III, ACA IgG III, and ACA IgA III; INOVA Diagnostics, Inc.). aPTT was determined using the Sta PTT Automate 5 and PTT-LA assay kits (Diagnostica Stago, Parsippany, NJ). dRVVT was determined by LA Screen and LA confirm assay kits (LASD-25 and LACD-10; Rainbow Scientific Inc., Windsor, CT). TTI was determined using the Sta Neoplastine CL +10 reagent (Diagnostica Stago).

The cutoff between positive and negative samples for all aPL assays was established as described in the respective kits. We defined a positive aPL test as a single measurement that fell outside the normal reference range: aCL (IgM ≥15.7 U/mL, IgG ≥19.3 U/ml, IgA ≥8.2 U/mL); anti-β2GPI (IgM ≥20 U/ml, IgG ≥20 U/ml, IgA ≥20 U/mL); and LA (aPTT ≥46 secs, dRVVT ≥40 secs, TTI ≥1.4).

### Statistical methods

Continuous variables, expressed as mean ± standard error, were compared by the median test or Wilcoxon rank-sum test. Categorical variables were compared by Fisher's exact test or McNemar's test between matched-paired data. SAS® software was used for statistical analyses and *P*<0.05 was considered statistically significant.

## Results

### Clinical characteristics

The majority of the 56 patients with isolated IgA anti-β2GPI were female (89%) and African-American (70%), with a mean age of 43 years. Thirty-one patients with isolated IgA anti-β2GP had SLE, while 25 did not. The titer of IgA anti-β2GPI was mildly elevated (20–50 U/mL) in 32 patients, moderately elevated (50–100 U/mL) in 15, and severely elevated (>100 U/mL) in 9. There were 38 total thromboembolic events (23 arterial, 10 venous, and 5 TMA) in 27 patients (48%), of whom 15 had SLE. Control individuals underwent complete aPL testing and were negative for all aPL. In controls, there were 18 total thromboembolic events (12 arterial, 6 venous) in 14 patients (25%), of whom 4 had SLE (Table 1). SLE disease duration did not differ between patients and controls (5.9±3.0 years vs. 6.8±4.8 years, p>0.5).

**Table 1:** Clinical characteristics of patients (stratified according to IgA anti-β2GPI titer) and controls.

	Patients with isolated IgA anti-β2GPI				Controls
	Mild elevation (20–49 U/mL)	Moderate elevation (50–99 U/mL)	Severe elevation (≥100 U/ml)	Total	Total
**Number of patients (%)**	32 (57%)	15 (27%)	9 (16%)	56 (100%)	56
**Age (range)**	43.3 years (22–78)	40.8 years (24–68)	44.4 years (30–61)	42.8 years (22–78)	42.8 years (22–78)
**Race**					
African-American	22	12	5	39	39
Asian	1	0	0	1	1
Caucasian	4	1	2	7	7
Hispanic	1	0	0	1	1
Unknown	4	2	2	8	8
**Gender**					
Female	30	13	7	50	50
Male	2	2	2	6	6
**Thromboembolic events** †					
Number of patients (%)	13 (41%)	10 (67%)	4 (44%)	27 (48%)	14 (25%)
Total Events	17	14	7	38	18
Arterial Events	10	8	5	23	12
Venous Events	5	3	2	10	6
TMA*	2	3	0	5	0
**SLE** * **status**					
SLE	16	11	4	31	32
Non-SLE#	16	4	5	25	24

*Abbreviations used in this table: SLE, systemic lupus erythematosus; TMA, thrombotic microangiopathy.

† Among IgA anti-β2GPI positive patients, 5 had both an arterial and a venous event (3 with mild elevation, 1 with moderate elevation, 1 with severe elevation), 4 patients had both an arterial event and TMA (2 with mild elevation, 2 with moderate elevation), 1 patient had both a venous event and TMA (with moderate elevation), and 1 patient had 2 arterial events (with severe elevation). Among controls, 4 had both an arterial and a venous event.

# Among IgA anti-β2GPI positive patients, non-SLE patients included the following autoimmune or rheumatologic diagnoses: Cogan's syndrome (*n* = 1), fibromyalgia (*n* = 1), Hashimoto's thyroiditis (*n* = 1), mixed connective tissue disease (*n* = 1), myelitis (*n* = 1), polycystic ovary syndrome (*n* = 1), sarcoidois (*n* = 1), scleroderma (*n* = 1), sickle cell anemia (*n* = 2), Sjögren's syndrome (*n* = 1), Tietze's syndrome (*n* = 1), and Wegener's granulomatosis (*n* = 1). Among controls, non-SLE individuals included: amyotrophic lateral sclerosis (*n* = 1), Behcet's syndrome (*n* = 1), chondrocalcinosis (*n* = 1), HELLP syndrome (*n* = 1), inflammatory polyarthropathy (*n* = 1), osteoporosis (*n* = 1), Raynaud's disease (*n* = 1), Sjögren's syndrome (*n* = 2), and ulcerative colitis (*n* = 1).

Twenty-three of the 56 patients with isolated IgA anti-β2GPI had repeat aPL testing, and, of these, 21 had persistently positive IgA anti-β2GPI. Thromboembolism occurred in 13 (62%) of the patients with persistently elevated IgA anti-β2GPI.

### Isolated IgA anti-β2GPI is associated with an increased risk of thromboembolic events

Isolated IgA anti-β2GPI was associated with an increased risk of thromboembolic events in the 56 patients with and without SLE (p = 0.018, odds ratio [OR] = 2.793, 95% confidence interval [CI] = 1.263–6.172). When we restricted our analysis to patients with SLE, isolated IgA anti-β2GPI was still associated with an increased risk (p = 0.026, OR = 4.282, 95% CI = 1.338–13.569). In contrast, among non-SLE patients, isolated IgA anti-β2GPI was not associated with an increased risk of thromboembolic events (p = 0.773, OR = 1.397, 95% CI = 0.456–4.276). These data (Table 2) indicate that IgA anti-β2GPI is associated with an increased risk of thromboembolic events in the presence, but not absence, of SLE.

**Table 2:** Prevalence of thromboembolic events and mucosal organ involvement in patients with isolated IgA anti-β2GPI versus controls.

Thromboembolic events	Isolated IgA anti-β2GPI	Controls	Odd ratio	95% CI	p value
***Entire cohort (n = 56)***					
≥1 thromboembolic event	27	14	2.793	1.263–6.172	0.018
No thromboembolic event	29	42			
***SLE (n = 31)***					
≥1 thromboembolic event	14	5	4.282	1.338–13.569	0.026
No thromboembolic event	17	26			
***Non-SLE (n = 25)***					
≥1 thromboembolic event	11	9	1.397	0.456–4.276	0.773
No thromboembolic event	14	16			
**Mucosal organ involvement**					
≥1 mucosal organs	49	37	3.595	1.394–9.223	0.013
No mucosal organs	7	19			

### Higher titers of IgA anti-β2GPI are associated with a greater risk for thromboembolic events in SLE patients

We next determined whether thromboembolic event occurrence correlated with higher median titers of IgA anti-β2GPI. Among patients with isolated IgA anti-β2GPI and SLE, higher titers were found in patients who had suffered a thromboembolic event (73.1±11.2 vs. 47.8±8.1 U/ml, p = 0.0262). Although a similar trend was observed among all patients with isolated IgA anti-β2GPI, it was not statistically significant (65.2±8.2 vs. 53.5±7.2 U/ml, p = 0.0926). No association between higher titers and thromboembolic event occurrence was found among non-SLE patients with isolated IgA anti-β2GPI (55.4±11.8 vs. 60.2±12.7 U/ml, p = 0.4253).

Among individuals with and without isolated IgA anti-β2GPI, there were no significant differences with respect to prevalence of hypertension, use of oral contraceptives, or level of proteinuria. Incomplete data within the medical records of patients and controls precluded analysis of other factors linked to thrombosis, such as smoking, homocycteine levels, other thrombophilic disorders, and hydroxychloroquine use.

### Isolated IgA anti-β2GPI is associated with an increased prevalence of co-morbidities involving the mucosal immune system

IgA antibodies are associated with disorders or diseases involving the organs of mucosal immunity, predominantly the GI system, pulmonary system, and skin. Secretion of IgA at the mucosal surface of these organs binds specifically to pathogens and inhibits their entry into the body. Isotype switching to IgA is induced by local factors produced within the lymphoid tissues of these organs.

**Figure 1:**
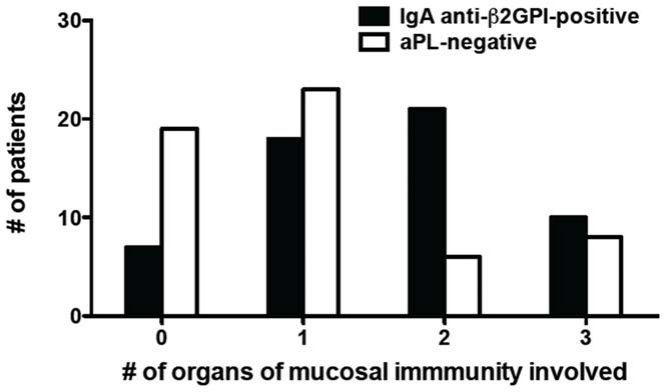
The presence of isolated IgA anti-β2GPI is associated with increased clinical involvement of the mucosal immune system (GI system, pulmonary system, or skin). Depicted are the total number of patients (IgA anti-β2GPI-positive) and controls (aPL-negative) who had a co-morbidity or disease involving 0, 1, 2, or 3 organs of mucosal immunity.

We therefore determined whether isolated IgA anti-β2GPI was associated with the presence of co-morbidities involving organs of mucosal immunity. Patients and matched controls were stratified according to the number of organs of mucosal immunity affected by at least one co-morbidity. Patients with isolated IgA anti-β2GPI typically had involvement of two or three mucosal organs, whereas the controls had fewer (no or one) co-morbidities (Figure 1) (p = 0.0021). When patients were stratified according to the presence or absence of at least one affected organ of mucosal immunity, patients with involvement of at least one mucosal organ were more likely to have isolated IgA anti-β2GPI antibodies (Table 2) (p = 0.0095, OR = 3.595, 95% CI = 1.394–9.223). Moreover, the mean number of mucosal organs affected by a co-morbidity was significantly greater among patients with isolated IgA anti-β2GPI (1.61±0.12 vs. 1.05±0.14, p = 0.007), as compared to those without isolated IgA anti-β2GPI. We also evaluated the association between isolated IgA anti-β2GPI and each individual organ of mucosal immunity. While there was a trend towards increased risk of involvement for each organ among patients with isolated IgA anti-β2GPI (lung: 46.4% vs. 28.6%, p = 0.078; GI: 44.6% vs. 28.6%, p = 0.116; skin: 62.5% vs. 48.2%, p = 0.183), the results did not achieve statistical significance. The specific comorbidities affecting each mucosal organ are given in Table 3.

**Table 3:** Comorbidities involving organs of mucosal immunity in patients with isolated IgA anti-β2GPI titer versus controls.

Comorbidities by Organ System	Patients with isolated IgA anti-β2GPI	Controls
**Gastrointestinal system**	**Total 25**	**Total 16**
GERD	14	11
Gastric/duodenal ulcers	4	0
Cholecystitis	2	0
Diarrhea	2	0
Hepatitis	1	0
Pancreatitis	1	2
Gastrointestinal bleed	1	1
IBD/ulcerative colitis	0	2
**Pulmonary system**	**Total of 26**	**Total 16**
Asthma	11	6
Interstitial lung disease	4	3
Pleuritis	3	0
Restrictive lung disease	2	0
Sleep apnea	2	0
Emphysema	1	0
Lung mass	1	0
Aspiration	1	0
Sarcoidosis	1	0
Bronchitis	0	1
COPD	0	3
Pulmonary hemorrhage	0	1
ARDS	0	1
Pleural effusion	0	1
**Skin**	**Total 35**	**Total 27**
Rash	15	11
Cutaneous lupus	7	9
Herpes zoster	5	2
Varicose veins	1	0
Livedo reticularis	1	0
Alopecia	1	0
Cutaneous ulcers	1	0
Skin cancer	1	1
Cutaneous sarcoid	1	0
Keloid	1	1
Vitiligo	1	0
Raynaud's	0	2
Psoriasis	0	1

*Abbreviations used in this table: ARDS, adult respiratory distress syndrome; COPD, chronic obstructive pulmonary disease; GERD, gastro-esophageal reflux disease; IBD, inflammatory bowel disease.

## Discussion

The utility of testing for IgA aPL is unclear and highly controversial. While some studies have found an association between IgA aPL and the clinical manifestations of APS [1], [2], [6], [7], [9], [10], [12]–[17], others have reported no association [4], [5], [8], [11], [18]. Here, we have determined whether IgA aPL are associated with clinical manifestations using an approach that differs from previous studies. We reasoned that if IgA aPL contribute to clinical manifestations of APS, then an association with thromboembolic events should manifest in patients whose only aPL is IgA. We limited our analysis to patients for whom IgA anti-β2GPI was the only aPL present. These patients and controls were also negative for IgA aCL. By restricting our analysis to isolated IgA anti-β2GPI, we limited the potentially confounding influence of other aPL specificities and isotopes on our results. A retrospective chart review of 56 patients with isolated IgA anti-β2GPI was performed, and the clinical features of these patients were compared with 56 individually matched control patients without aPL.

Our results add to the growing evidence that patients with IgA aPL are at increased risk for thromboembolic events. First, we found that patients with isolated IgA anti-β2GPI had significantly more thromboembolic events than controls. Second, among patients with isolated IgA anti-β2GPI, those with at least one thromboembolic event had significantly higher titers of IgA anti-β2GPI than patients without a thromboembolic event. Third, isolated IgA anti-β2GPI presence was associated with an increased prevalence of diseases or morbidities involving organs of mucosal immunity. Finally, when patients were stratified into those with and without SLE, the association between isolated IgA anti-β2GPI and thromboembolic events persisted for patients with SLE, but was lost for those without SLE.

The lack of association between isolated IgA anti-β2GPI and thromboembolic events among non-SLE patients requires comment. Two methodological issues may have contributed to this negative result. The first is our limited study size. Insufficient power may have precluded detection of an association among non-SLE patients, especially if the risk conferred by isolated IgA anti-β2GPI is less for non-SLE than SLE patients. The second issue relates to a potential bias in non-SLE patient selection. Although patients and controls were selected on the basis of laboratory records for aPL testing and individually matched, the rationale for aPL testing may have differed for SLE versus non-SLE patients. For SLE patients, aPL testing is typically conducted within a screen for SLE-associated autoantibodies. In contrast, for non-SLE patients, aPL testing would more likely be performed because of suspected hypercoagulability. Thus, non-SLE patients lacking IgA anti-β2GPI may have been biased towards an increased prevalence of thromboembolic events (i.e., the outcome measured). This potential bias is apparent when comparing the frequency of patients with at least one thromboembolic event in non-SLE versus SLE controls (i.e., patients lacking aPL) (non-SLE: 36% vs. SLE: 16%). In contrast, among patients with isolated IgA anti-β2GPI, the frequency of individuals with at least one thromboembolic event is more closely matched in non-SLE versus SLE patients (non-SLE: 44% vs. SLE: 45%), suggesting the predominating effect of aPL.

There are several reasons why the concomitant presence of SLE may increase the risk of thromboembolism in patients with isolated IgA anti-β2GPI. First, SLE may confer an increased risk for thrombosis that is independent from that of aPL [21], [22]. Second, concurrent inflammation and activation of innate immunity during flares of SLE may provide a “second hit” necessary for aPL to induce thrombosis [23]. Third, the epitope specificity of IgA anti-β2GPI may differ depending upon whether aPL arise in the presence or absence of systemic autoimmunity. For example, among anti-β2GPI, antibodies that recognize domain I of β2GPI are predictive of thrombosis, whereas those that recognized other domains are not [24]. Finally, other autoantibodies found in SLE patients, such as anti-heat shock protein 60 antibodies, may interact with aPL to enhance thrombotic potential [25], [26].

Our study has several limitations, in addition to its small sample size and possible selection bias for non-SLE patients. First, the retrospective nature of our study prevented us from obtaining complete data on several non-aPL-related risk factors for thrombosis, such as smoking, homocycteine levels, and other thrombophilic disorders. In addition, many patients did not undergo repeat testing for aPL, so we could not determine if IgA anti-β2GPI were transient or persistent.

In conclusion, our data indicate that IgA anti-β2GPI is associated with an increased risk of thromboembolic events in patients with SLE. Our data do not permit a definite conclusion regarding the role of IgA anti-β2GPI in patients without SLE. We also report that the presence of IgA anti-β2GPI is associated with co-morbidities affecting organs involved in mucosal immunity. Prospective studies of patients with IgA anti-β2GPI, isolated or in association with other aPL, are needed to clarify the role of IgA anti-β2GPI as a risk factor for thrombosis.
